# Gender Differences in the Progression of Experimental Chronic Kidney Disease Induced by Chronic Nitric Oxide Inhibition

**DOI:** 10.1155/2017/2159739

**Published:** 2017-10-18

**Authors:** Camilla Fanelli, Humberto Dellê, Rita Cassia Cavaglieri, Wagner Vasques Dominguez, Irene L. Noronha

**Affiliations:** ^1^Laboratory of Cellular, Genetic, and Molecular Nephrology, Renal Division, University of São Paulo, São Paulo, SP, Brazil; ^2^Laboratory of Renal Pathophysiology, University of São Paulo Medical School, São Paulo, SP, Brazil; ^3^NETCEM (Cell and Molecular Therapy Center), University of São Paulo, São Paulo, SP, Brazil

## Abstract

Chronic kidney disease (CKD) is considered a public health problem, assuming epidemic proportions worldwide. In this context, the preponderance of CKD prevalence in male over age-matched female patients is of note. In the present study, we investigated the impact of the gender on the development of experimental CKD induced by chronic nitric oxide (NO) inhibition in Wistar male and female rats through the administration of L-NAME. CKD model induced by L-NAME is characterized by systemic vasoconstriction, resulting in severe hypertension, albuminuria, renal ischemia, glomerulosclerosis, interstitial expansion, and macrophage infiltration. After 30 days of CKD induction, male NAME rats exhibited remarkable albuminuria, augmented cortical histological damage, interstitial inflammation, and fibrosis. Age-matched female NAME rats showed significantly lower albuminuria, diminished glomerular ischemia, and glomerulosclerosis, as well as a significant reduction in the expression of *α*-smooth muscle actin renal interstitial Ang II^+^ cells. Thus, the present study demonstrated that female rats submitted to the NAME model developed less severe CKD than males. Female renoprotection could be promoted by both the estrogen anti-inflammatory activity and/or by the lack of testosterone, related to renin-angiotensin-aldosterone system hyperactivation and fibrogenesis. However, the influence of sex hormones on the progression of CKD needs to be further investigated.

## 1. Introduction

In the last decades, chronic kidney disease (CKD) became a public health problem with epidemic proportions worldwide, with more than 2 million of patients requiring hemodialysis or other renal replacement therapy [1, 2]. The progressive feature of CKD represents an additional reason for addressing this disease as a serious public health concern nowadays. In this context, the well-recognized preponderance of CKD prevalence in male over female patients is of note. According to the United States Renal Data System (USRDS), 62% of CKD patients that reached end-stage renal failure in 2015 were men, whereas only 38% were women [3]. In addition, it has been previously shown that women with CKD have a slower decline in renal function with time when compared with men [4–6].

A higher incidence and prevalence of renal failure particularly in nondiabetic renal patients is observed in men. According to Silbiger and Neugarten, the ratio between men and women that reaches renal insufficiency due to hypertensive nephropathy or glomerulonephritis is 1.6 men for each woman affected [5]. Similarly, there are more than 2 men affected by IgA nephropathy or membranous nephropathy for each woman [7]. Data derived from the Dialysis Outcomes and Practice Patterns Study (DOPPS) showed that male CKD patients are prone to start dialysis earlier than women and usually exhibit a worse survival rate [8]. The possible protective role of estrogens on the progression of renal disease is further evidenced by the lower prevalence of CKD in premenopausal women compared with age-matched men. Interestingly, this renoprotection observed in females disappears after the beginning of menopause, when the estrogen serum levels decreases drastically, suggesting an important role of sexual hormones on the development of renal function loss [8–10].

It is still not clear whether the lower prevalence of CKD in females is due to a hormonal renoprotection promoted by estrogens or due to the absence of the profibrotic effects caused by testosterone [5, 6]. Lombet et al. have shown that male rats submitted to the 5/6 renal ablation model exhibited a reduction of the progressive course of CKD after castration [11]. Corroborating these findings, Tomiyoshi et al. showed that castration attenuated proteinuria and glomerulosclerosis and ameliorated glucose tolerance in male spontaneously hyperglycemic Otsuka Long-Evans Tokushima Fatty (OLETF) rats [12]. More recently, Verzola and collaborators demonstrated that testosterone stimulates tubular epithelial cell apoptosis, leading to renal damage [13].

Regardless of the gender, it is well known that the process that leads to end-stage renal failure can be triggered by both immunological and nonimmunological events, such as glomerulonephritis, hypertensive nephrosclerosis, or diabetes mellitus. Irrespective of the nature of the initial renal insult, the progression of CKD is closely related to the renin-angiotensin system (RAS) overactivity. Angiotensin II (Ang II) overexpression promotes vasoconstriction, increased tubular sodium reabsorption, hypertension, and renal inflammation, characterized by interstitial macrophage/lymphocyte infiltration and cellular proliferation followed by an increase on extracellular matrix (ECM) deposition, loss of histological architecture, glomerulosclerosis, and interstitial fibrosis, which culminates in chronic renal insufficiency [14, 15].

In the present study, we seek to investigate the impact of the gender on the development of chronic experimental progressive kidney disease. For this purpose, male and female Wistar rats were submitted to the NAME experimental CKD model, in which renal disease is induced by chronic nitric oxide (NO) inhibition through the administration of a L-arginine analogue, N^*ω*^-nitro-L-arginine methyl-ester (L-NAME), associated with a high-salt diet [16, 17]. NO is a gaseous reactive oxygen species synthesized by the oxidation of L-arginine to L-citrulline, catalyzed by a group of NO synthases (NOS) which can be either inducible (iNOS) or constitutive (cNOS). The inducible isoforms of NOS are commonly seen in monocytes, macrophages, and other cytotoxic immune cells. However, different cell types can express iNOS and produce NO when stimulated by proinflammatory cytokines. In these cells NO exerts a nonspecific microbicidal effect. On the other hand, constitutive NOS are present in both neuronal (nNOS) and endothelial (eNOS) cells, in which NO is required to maintain the integrity of nerves and to act as a potent vasodilator, respectively. The CKD model induced by NAME is characterized by progressive systemic vasoconstriction, resulting in hypertension, albuminuria, glomerular sclerosis and ischemia, interstitial expansion, and progressive macrophage infiltration characterized by progressive systemic vasoconstriction, resulting in hypertension, albuminuria, glomerular sclerosis and ischemia, interstitial expansion, and progressive macrophage infiltration [16, 17].

The aim of the present study was to investigate the main gender differences on the progression of the NO chronic inhibition nephropathy in male and female Wistar rats.

## 2. Methods

### 2.1. Experimental Groups

The NAME experimental model was obtained by diary oral administration of an L-arginine analogue, N^*ω*^-nitro-L-arginine methyl-ester (L-NAME, Sigma Chemical Co., St. Louis, USA), 200 mg/L, diluted in drinking water, associated with high-sodium (HS) diet (3.12% Na, Nuvital, BR). Twenty male and 20 female Wistar rats aging between 7 and 8 weeks were obtained from an established colony at the University of São Paulo, Brazil. The animals were maintained at 23 ± 1°C on a 12/12-hour light/dark cycle with free access to tap water and HS diet ad libitum. After 2 weeks of adaptation to HS diet, rats were divided among 4 groups: Control male (*Control *** ♂**) and Control female (*Control *** ♀**), receiving only HS diet; NAME male (*NAME *** ♂**) and NAME female (*NAME *** ♀**), receiving HS and L-NAME.

### 2.2. Long-Term Studies

Rats were followed up for 30 days of treatment and their body weight was analyzed weekly. By the end of this period, blood pressure was measured by evaluating their tail-cuff pressure (TCP) measured by an indirect method (RTBP 2045; Kent Scientific). The animals were, then, placed in metabolic cages for determination of 24-hour urinary albumin excretion rate (24 h UAE) by radial immunodiffusion, expressed by mg/24 h [18]. Animals were, thereafter, anesthetized with sodium pentobarbital, 60 mg/kg intraperitoneal (IP), and submitted to total nephrectomy followed by euthanasia through overdose of sodium pentobarbital, 80 mg/kg IP.

All the experimental procedures performed in this study were approved by the local Research Ethics Committee (CAPPesq, process number 796/00) and were developed in strict conformity with our institutional guidelines and with international standards for manipulation and care of laboratory animals. All rats were manipulated daily for monitoring their general health condition.

### 2.3. Histological Analysis

Kidneys obtained from total nephrectomy were cut in two midcoronal renal slices and previously treated with Duboscq-Brazil, during 30 minutes. Renal fragments were, then, postfixed in buffered 4% formaldehyde and embedded in paraffin using conventional sequential techniques. Finally, 4 *μ*m thick tissue sections were obtained and examined for assessment of glomerular and tubulointerstitial remodeling.

In order to evaluate the presence of glomerular damage, the percentage of sclerotic glomeruli (glomerulosclerosis) and the percentage of ischemic (collapsed) glomeruli found in at least 50 randomly sampled glomerular tuft profiles per rat were evaluated in periodic acid-Schiff (PAS) staining samples. Glomerulosclerosis was defined as segmental hyalinosis lesions, usually with adhesion to Bowman's capsule. Collapsed glomeruli were defined as glomeruli reduced in size and exhibiting a wrinkling of the basement membrane with capillary loops uniformly collapsed.

The extent of interstitial expansion reflecting fibrotic regions was quantitatively evaluated in Masson-stained sections by a point counting technique [19].

### 2.4. Immunohistochemistry/Immunofluorescence

Immunohistochemistry (IHC) assays were performed to identify interstitial macrophage and T-cell infiltration in renal sections, as well as to quantify the percentage of tubulointerstitial area occupied by *α*-smooth muscle actin (*α*-SMA), a marker of myofibroblasts, considered as effector cells of fibrogenesis. Finally, we also accessed the presence of renal interstitial cells positive for Ang II through immunohistochemistry as well [15]. Additionally, double-staining immunofluorescence was performed in order to verify if infiltrating macrophages and/or myofibroblasts were the responsible for interstitial Ang II production in NAME male and female rats.

For specific immunostaining of macrophages, a mouse monoclonal anti-rat ED1 antibody (Serotec, #MCA341R, Oxford, UK) was used and APAAP (alkaline phosphatase-antialkaline phosphatase) IHC technique was applied. Monoclonal mouse anti-CD3 (Dako, #M7254, ‎Glostrup, ‎Denmark) and anti-*α*-SMA (Sigma Chemical CO, #A2547, St. Louis, USA) and a rabbit polyclonal anti-angiotensin II (Peninsula Lab. Int., #T4007, CA, USA) antibodies were used, respectively, to identify T-cells, myofibroblasts, and angiotensin II, by means of a streptavidin-biotin-alkaline-phosphatase IHC technique. Reactions were developed with a fast-red dye solution, and counterstained with Mayer's haemalum (Merck, Darmstadt, Germany), as described in previous studies [20, 21]. For double-staining immunofluorescence sections were deparaffinized, microwaved for antigen retrieval, subjected to nonspecific blocking, and incubated overnight at 4°C with the same anti-ED1, anti-*α*-SMA, and anti-angiotensin II primary antibodies employed for immunohistochemistry. The sections were, then, incubated with the secondary antibodies Alexa Fluor 633 goat anti-mouse and Alexa Fluor 488 donkey anti-rabbit (Thermo Fisher Scientific) and finally with the blue-fluorescent DNA stain DAPI (4′, 6-diamidino-2-phenylindole). Slides were cover slipped with glycerin solution and analyzed using optical microscopy with dark field (Olympus-BX51, Tokyo, Japan) under ×400 magnification and analyzed with the image-processing software (Image Pro Plus, version 7.01).

### 2.5. Statistical Analysis

Results are presented as mean ± SEM. One-way analysis of variance with pairwise comparisons according to the Newman-Keuls method was used to compare experimental groups. All calculations were realized by the GraphPad Prism 2.01 software (^a^*p* < 0.05 versus Control ♂; ^b^*p* < 0.05 versus Control ♀; ^c^*p* < 0.05 versus NAME ♂) [22].

## 3. Results

### 3.1. Experimental CKD Induced by L-NAME Promoted Lower Albuminuria in Female Than in Male Rats

**Figure 1:**
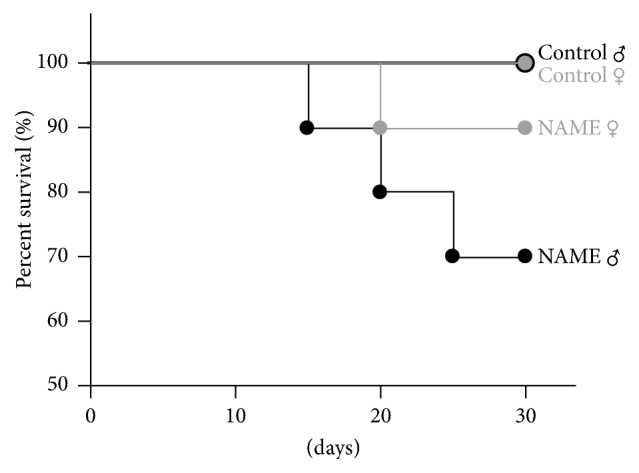
Survival rates of both male and female Control and NAME groups. In this study, 10 animals were included in each experimental group (*n* = 40). Three male and one female NAME-treated rats died before the end of the study, whereas no mortality was observed in Control groups, regardless of the gender.

Both male and female rats with CKD induced by L-NAME administration exhibited significantly lower body weight compared with Control groups (Table 1). However, there was no difference in the growth rate between male and female NAME rats induced by L-NAME. In this study, 20 male and 20 female Wistar rats were included (10 male and 10 female animals received L-NAME associated with HS diet and 10 male and 10 female Control animals received only tap water and HS diet). Three male and only one female NAME-treated animals died before the end of the study period (30 days), whereas no mortality was observed in the Control groups, regardless of the gender (Figure 1).

Rats receiving L-NAME developed severe hypertension. Tail-cuff pressure was markedly elevated in NAME rats, both in male (210 ± 11 versus 127 ± 1 mmHg in Control ♂; *p* < 0.05) and in female rats (239 ± 10 versus 131 ± 5 mmHg in Control ♀; *p* < 0.05). (Table 1). Additionally, the 24 h UAE was also strikingly increased in NAME rats, both male (118.71 ± 36.20 versus 1.42 ± 0.50 mg/24 h in Control ♂; *p* < 0.05) and female (42.81 ± 23.26 versus 1.41 ± 0.48 mg/24 h in Control ♀; *p* < 0.05) (Table 1). However, it is of note that NAME ♀ presented a significantly higher elevation of blood pressure (239 ± 10 versus 210 ± 11 mmHg in NAME ♂; *p* < 0.05) but significantly lower 24 UAE than male rats exposed to the same experimental model of CKD (42.81 ± 23.26 versus 118.71 ± 36.20 mg/24 h in NAME ♂).

### 3.2. Glomerular Damage Protection in Female Rats Submitted to L-NAME-Induced CKD

**Figure 2:**
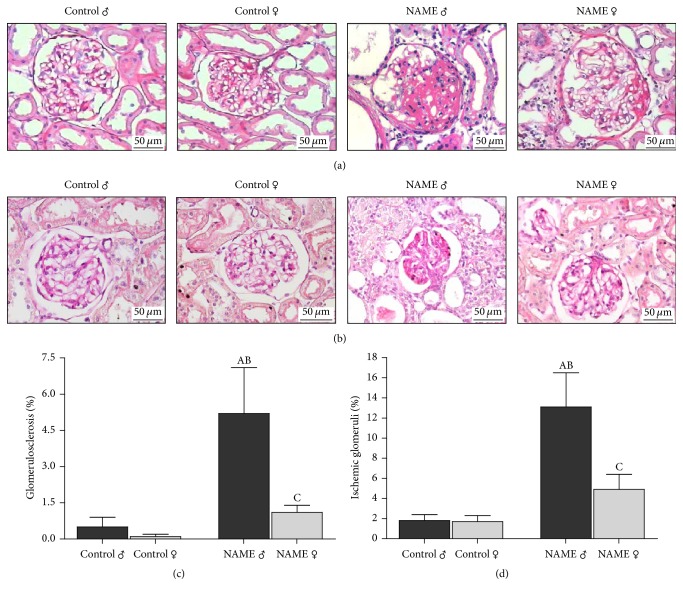
Representative micrographs of glomerulosclerosis (a) and collapsed glomeruli (b) accessed by PAS staining in groups Control and NAME, male and female. Male NAME rats developed a marked degree of glomerulosclerosis, whereas female NAME rats exhibited a protection of this renal damage feature (a). Glomerular ischemia, represented by the presence of collapsed glomeruli, was also observed in NAME rats, with a higher degree in male than in female animals (b). Accordingly, bar graph representation of percentage of glomerulosclerosis (c) and glomerular ischemia (d) in groups Control and NAME showed that female NAME rats presented renoprotection compared to male NAME animals (^A^*p* < 0.05 versus Control ♂, ^B^*p* < 0.05 versus Control ♀, ^C^*p* < 0.05 versus NAME ♂). The number of animals studied per group was, respectively, Control ♂: *N* = 10; Control ♀: *N* = 10; NAME ♂: *N* = 7; NAME ♀: *N* = 9.

The analysis of glomerular structural damage, characterized by the development of glomerulosclerosis, and glomerular ischemia in the NAME experimental CKD model induced in male and female rats is shown in Figure 2. Loss of regular glomerular architecture was seen in both male and female NAME animals. However, it is of note that these alterations were statistically significant only in male NAME rats, which developed severe glomerulosclerosis (5.2 ± 1.9 versus 0.5 ± 0.4% in Control ♂; *p* < 0.05) and glomerular ischemia (13.1 ± 3.4 versus 1.8 ± 0.6% in Control ♂; *p* < 0.05) when compared to their gender matched Controls. Female NAME rats presented only numerical increases in the percent of both sclerotic (1.1 ± 0.3 versus 0.1 ± 0.1% in Control ♀; *p* > 0.05) and ischemic (4.9 ± 1.5 versus 1.7 ± 0.6% in Control ♀; *p* > 0.05) glomeruli, reflecting female renoprotection in this model. Moreover, glomerular structural injury was significantly lower in female than in male NAME animals, demonstrating that female animals were resistant to the development of glomerulosclerosis and ischemia, even in a setting of systemic hypertension with higher blood pressure levels in female compared with male rats.

### 3.3. Myofibroblast Infiltration, But Not Interstitial Expansion, Is Blocked in Female Rats Submitted to the L-NAME-Induced CKD Model

**Figure 3:**
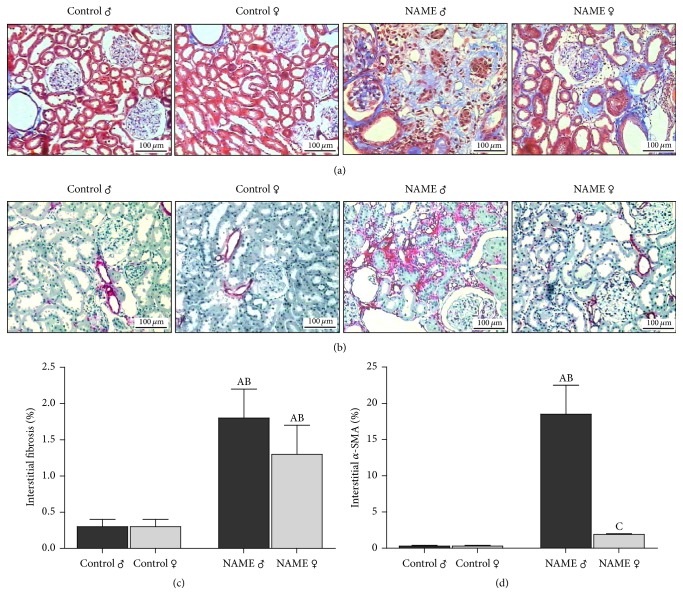
Representative micrographs of renal cortical interstitial fibrosis (a), evaluated in Masson's trichrome stained slides, and interstitial *α*-SMA accumulation (b), accessed by immunohistochemistry. Prominent interstitial expansion was observed in both male and female NAME animals, while significant accumulation of interstitial *α*SMA was seen only in male NAME rats. Accordingly, bar graph representation of percentage of cortical interstitial fibrosis (c) and interstitial *α*-SMA accumulation (d) showed that female animals submitted to the NAME CKD model were protected from interstitial myofibroblast infiltration when compared to male NAME rats (^A^*p* < 0.05 versus Control ♂, ^B^*p* < 0.05 versus Control ♀, ^C^*p* < 0.05 versus NAME ♂). The number of animals studied per group was, respectively, Control ♂: *N* = 5; Control ♀: *N* = 5; NAME ♂: *N* = 5; NAME ♀: *N* = 5.

As can be seen in Figure 3, kidney samples stained with Masson's trichrome revealed prominent renal interstitial expansion in male (1.8 ± 0.4%, *p* < 0.05, versus 0.3 ± 0.1% in Control ♂) and to a less degree in female NAME rats (1.3 ± 0.4%, *p* < 0.05, versus 0.3 ± 0.1%, in Control ♀). However, the analysis of *α*-SMA, a common marker of myofibroblasts, considered as effector cells of fibrogenesis, showed a striking different expression between male and female NAME rats. Male rats submitted to the NAME-induced CKD model presented a marked number of interstitial myofibroblasts in the kidney. In contrast, in female NAME rats, the expression of interstitial *α*-SMA, reflecting the presence of myofibroblasts was blocked.

### 3.4. Rats Submitted to the L-NAME-Induced CKD Model Developed Inflammation Regardless of the Gender

**Figure 4:**
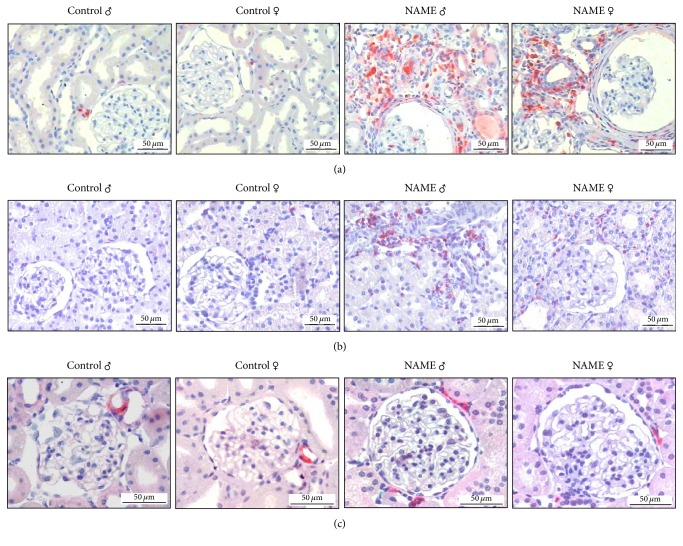
Representative micrographs of immunohistochemical studies in groups Control and NAME: both male and female NAME animals showed a significant increase of renal interstitial macrophage (a) and T-cell (b) infiltration. Additionally, male NAME rats exhibited a statistically increased number of interstitial Ang II^+^ cells, compared to gender matched Controls (c).

**Figure 5:**
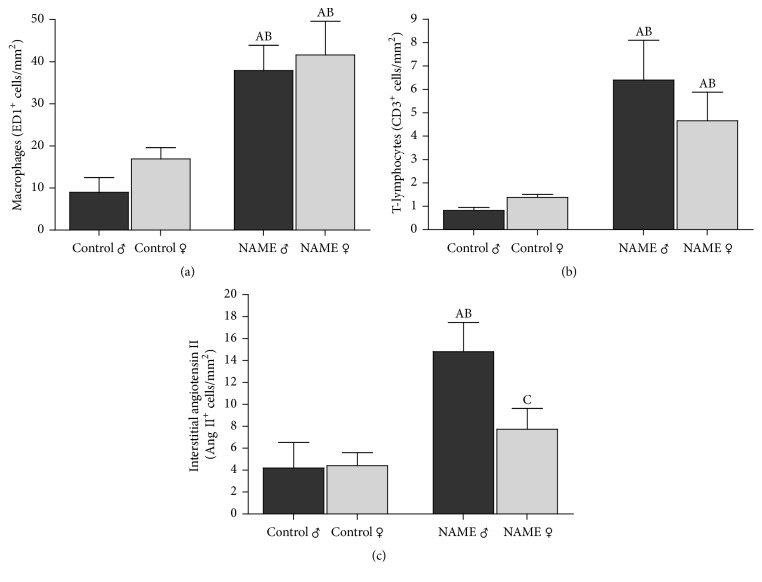
Bar graph representation of renal cortical interstitial infiltration by macrophages (a), T-cells (b), and Ang II*α*^+^ cells (c) showed that both male and female NAME rats exhibited a significant increase of renal macrophage and T-cell infiltration. However, NAME females showed statistically less Ang II^+^ interstitial cells than males subjected to the same treatment (^A^*p* < 0.05 versus Control ♂, ^B^*p* < 0.05 versus Control ♀, ^C^*p* < 0.05 versus NAME ♂). The number of animals studied per group was, respectively, Control ♂: *N* = 5; Control ♀: *N* = 5; NAME ♂: *N* = 5; NAME ♀: *N* = 5.

NAME rats exhibited a significant increase of interstitial macrophage infiltration (Figures 4(a) and 5(a)), regardless of the gender. The number of ED1^+^ interstitial cells was significantly high in both male and female NAME rats (37.9 ± 6.0 and 41.6 ± 8.0, resp., compared to 9.0 ± 3.5 in Control ♂ and 16.9 ± 2.7 in Control ♀; *p* < 0.05). Similar results were obtained for renal interstitial T-cell infiltration (Figures 4(b) and 5(b)). Both male and female NAME rats showed a significantly increased number of interstitial T-cells when compared to their gender matched Controls (6.4 ± 1.7 and 4.7 ± 1.2, resp., compared to 0.8 ± 0.1 in Control ♂ and 1.4 ± 0.1 in Control ♀; *p* < 0.05).

### 3.5. Renal Interstitial Ang II^+^ Cells Are Reduced in Female Compared to Male NAME Rats

In the kidney specimens obtained from Control rats that received high-salt diet, Ang II was detected almost exclusively in glomerular arterioles. However, in NAME rats, Ang II expression was detected in the afferent arterioles and also in mononuclear interstitial cells. The number of positively stained cells for Ang II in cortical interstitial cells of male NAME rats was significantly higher than in male Controls (15 ± 3, *p* < 0.05, versus 4 ± 2, in Control ♂), as shown in Figures 4(c) and 5(c). Alternatively, in female NAME animals, the number of interstitial cells staining positively for Ang II was not statistically different from the observed in Control group (8 ± 2, *p* > 0.05, versus 4 ± 1, in Control ♀). Moreover, interstitial Ang II^+^ cell infiltration was significantly higher in male than in female NAME rats (8 ± 2, *p* > 0.05, versus 15 ± 3 in NAME ♂).

**Figure 6:**
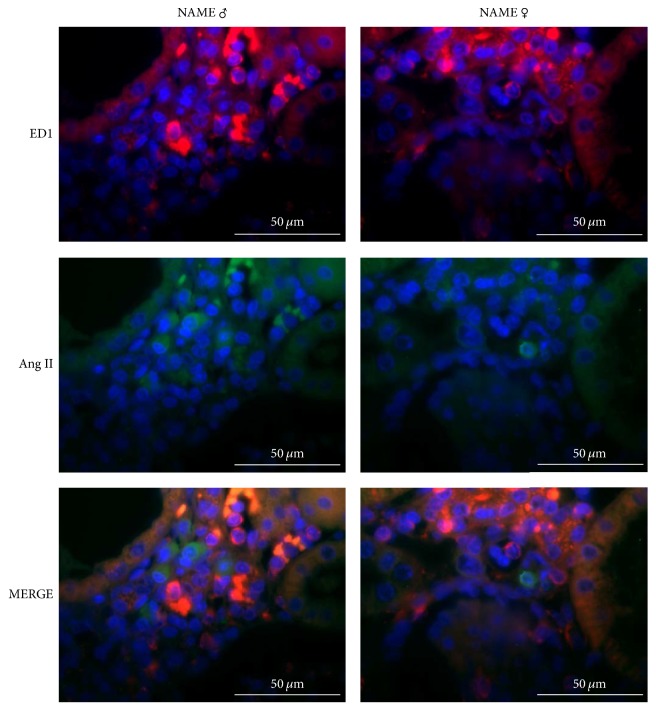
Representative micrographs of immunofluorescence double-staining for macrophages (ED1) and angiotensin II (Ang II) in NAME male and female animals. There was no evidence of colocalization of both biomarkers in the same cell (MERGE).

**Figure 7:**
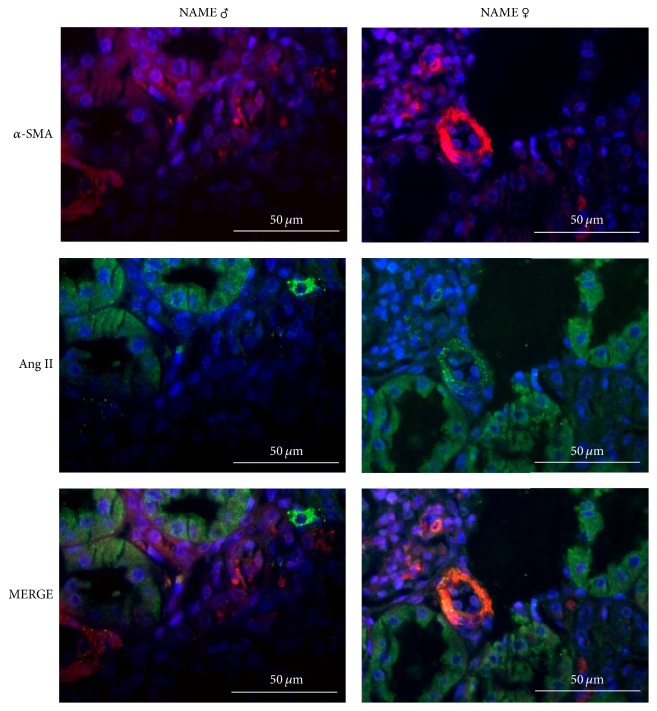
Representative micrographs of immunofluorescence double-staining for myofibroblasts (*α*-SMA) and angiotensin II (Ang II) in NAME male and female animals. There was no evidence of colocalization of both biomarkers in interstitial cells. Colocalization was only constitutively observed in glomerular arterioles and mesangial cells (MERGE).

In order to verify which kind of inflammatory cell could be the responsible for interstitial Ang II positivity in NAME rats, we performed additional immunofluorescence double-staining for both ED1 and Ang II and *α*-SMA and Ang II. As illustrated in Figure 6, interstitial Ang II^+^ cells were apparently not macrophages, since we could not observe any colocalization of ED1 and Ang II in the renal cortex of both male and female NAME rats. Moreover, interstitial myofibroblasts also seem not to be responsible for interstitial Ang II positivity in the renal parenchyma of NAME rats, since we found *α*-SMA and Ang II colocalization only in glomerular arterioles and mesangial cells, renal sites in which both proteins are predicted to be, even under physiologic conditions. There were no interstitial cells staining positively for both *α*-SMA and Ang II simultaneously (Figure 7).

## 4. Discussion

In the present study, NO synthesis was chronically inhibited by the administration of L-NAME, an L-arginine analogue, in Wistar male and female rats. Corroborating previous findings, animals treated with L-NAME presented poor weight gain and severe hypertension [16, 17]. Interestingly, we observed that female Wistar rats submitted to the NAME model developed significantly higher blood pressure levels than male rats. Verhagen and collaborators, using lower doses of L-NAME, reported a dose-dependent increase in blood pressure but no difference between males and females [23]. This difference may be accounted by the lower doses of L-NAME employed by these authors, which caused lower blood pressure levels than observed in the present study.

As previously described, chronic inhibition of L-NAME promotes increased urinary albumin excretion, reflecting a disruption of the glomerular filtration barrier [24]. In the present study, we observed significantly higher urinary albumin excretion levels in male rats than females exposed to the same experimental model of CKD. These results may be explained by the effect of androgens inducing higher susceptibility to proteinuria in male rats submitted to the L-NAME model, since orchidectomy has been shown to prevent the development of proteinuria in NAME rats [23]. Curiously, female NAME rats, despite presenting pronouncedly high blood pressure levels, did not develop significant albuminuria compared with male NAME rats. This apparent discrepancy between hypertension and the absence of abnormal urinary albumin excretion may be explained by the protective effect of estrogens in females, as described by Verhagen et al. [23]. In other models of renal disease, there is also evidence that estrogen or estrogen metabolites exert renoprotective effects, reducing albuminuria, attenuating renal lesions, and preventing CKD progression [25, 26]. The beneficial effects of estrogens in the kidney are possibly related to their known effects on mesangial cells [27] and on podocytes [28]. Moreover, in an elegant study focused on the molecular determinants of albuminuria, Long and collaborators demonstrated that a reduced number of big glomeruli associated with high levels of circulating testosterone are potential mechanisms leading to albuminuria in mice [29].

Chronic inhibition of NO, a remarkable physiological vasodilator, induces systemic vasoconstriction with deleterious renal hemodynamic effects, characterized by renal ischemia and collapsed glomeruli [16, 17]. In the present study, a significant higher degree of collapsed glomeruli was observed in male NAME rats compared with female animals. Previous studies have shown that the renal vasculature of male animals are likely more dependent on NO than that of females [30]. The protective effect observed in female rats may be related to the role of estrogen in improving both eNOS and iNOS activity, optimizing their affinity for L-arginine, thus attenuating the effects of L-NAME administration [31].

Female NAME rats were also more resistant to the development of glomerulosclerosis, even in a setting of systemic hypertension with striking high blood pressure levels. Previous studies have described the potential renoprotective effects of estrogen in the kidney by decreasing mesangial cell proliferation as well as decreasing the synthesis and accumulation of mesangial ECM, thus reducing the incidence of glomerulosclerosis [25, 26, 28, 32–34]. Moreover, a reduction of the steady mRNA message for the *α*-2 chain of type I collagen, inhibiting collagen synthesis, has been demonstrated in mesangial cells* in vitro* [27]. Estrogen has also been shown to reduce TGF-ß expression, which could explain the antifibrotic effects [35–37].

Both male and female rats chronic submitted to the progressive nephropathy model induced by inhibition of NO synthesis presented marked cortical interstitial expansion stained with Masson's trichrome, reflecting interstitial fibrosis. However, the expression of *α*-SMA, a common marker of myofibroblasts, considered as effector cells of fibrogenesis, was remarkably increased in male NAME rats, but not in females, strongly suggesting the presence of myofibroblasts in the renal parenchyma of male animals [38]. Myofibroblasts can originate directly from resident fibroblasts or by transdifferentiation from tubular epithelial cells (epithelial-to-mesenchymal transition). Regardless of their origin, these cells can synthesize proinflammatory cytokines and growth factors leading to the recruitment of inflammatory cells and ECM protein production, contributing to fibrogenesis [15, 38]. The absence of interstitial myofibroblasts in the renal cortex of female NAME rats indicates that the progressive fibrogenesis that parallels CKD progression, responsible for the gradual renal function loss, is abrogated in female animals, suggesting that the female gender presents renoprotection features. Our findings are consistent with the observations of Tostes et al. that estrogen exerts a direct inhibitory effect on smooth muscle cells by activating potassium efflux, inhibiting calcium influx and reducing vascular smooth muscle cell proliferation* in vitro* [39].

Tubulointerstitial inflammation, characterized by macrophage and lymphocyte infiltration, was observed in the kidney of both male and female NAME rats, suggesting that the possible renoprotective effects of sexual hormones do not rely on the inflammatory axis implicated in the progression of CKD. On the other hand, renal interstitial cells expressing Ang II were detected in animals submitted to the NAME model. We have previously reported the renal expression of Ang II and all other components of RAS in NAME rats, demonstrating an upregulation of intrarenal RAS in this model [20]. The pathogenetic role of intrarenal generated Ang II is not clear but the unusual expression of Ang II in renal interstitial cells may be related to mechanisms involved in the progression of renal disease, particularly through proinflammatory and profibrotic pathways [40–43]. The intrarenal RAS seems to have an independent regulation to the systemic endocrine RAS [41]. Despite the intense vasoconstriction induced by the blockade of NO synthesis, the analysis of systemic RAS in this model showed no difference in plasma renin activity and plasma Ang II levels [20], possibly due to the high-salt diet, which shuts off renin release, counterbalancing the strong ischemic stimulus to renin secretion [20].

In our experiments, Ang II expression detected in interstitial cells in the kidney was markedly lower in female than in male NAME rats, suggesting a downregulation of the intrarenal RAS in female NAME rats. These results are in line with previous findings of other laboratories that showed a possible interaction between estrogen and the RAS activity. Brosnihan and collaborators demonstrated that estrogen protects transgenic hypertensive rats by shifting the vasoconstrictor-vasodilator balance of RAS [44]. Studies developed by Komukai et al. indicated that female sex hormone decreased renin levels, angiotensin-converting enzyme (ACE) activity, Ang II receptor-1 expression, and aldosterone production, thus contributing to renoprotection against glomerular damage and inflammation [45]. On the other hand, Katz and Roper demonstrated that testosterone could stimulate RAS activity [46], which can also represent a reasonable explanation for the modest glomerular injury observed in female NAME rats, since their testosterone plasma levels are about tenfold lower than those observed in adult male rats.

In the present study, we attempted to elucidate the interstitial cell type expressing Ang II. The results obtained with the double-staining experiments for ED1 (macrophages), *α*-SMA (myofibroblasts), did not show colocalization of ED1 and Ang II nor *α*-SMA and Ang II, indicating that the cells sources are neither macrophages nor myofibroblasts.

In summary, our data show gender differences in the progression of experimental chronic kidney disease induced by chronic NO inhibition. Male NAME rats showed significantly higher albuminuria levels, augmented cortical histological damage, accumulation of myofibroblasts in the interstitium, and higher number of renal interstitial Ang II^+^ cells compared with female animals submitted to the same model. It is not clear whether the increased severity of renal damage parameters in males compared with females are due to the elevated serum testosterone concentrations or to the reduced estrogen serum content. The combination of low testosterone and high estrogen plasma levels could be, at least partially, responsible for the exuberant renoprotection exhibited by NAME females rats compared with male animals. Additional studies are required to investigate whether sexual hormones can dictate gender differences in CKD progression.

## Figures and Tables

**Table 1:** Functional and renal parameters observed after 30 days of treatment.

	Control ♂	Control ♀	NAME ♂	NAME ♀
BW (g)	336 ± 7	215 ± 4^a^	269 ± 13^a^	221 ± 3^a^
ΔBWG	31 ± 9	27 ± 1	4 ± 7	4 ± 1^ab^
BP (mmHg)	127 ± 1	131 ± 5	210 ± 11^ab^	239 ± 10^abc^
24 h UAE (mg/24 h)	1.4 ± 0.5	1.4 ± 0.5	118.7 ± 36.2^ab^	42.8 ± 23.0^c^

Values are presented as mean ± SEM for body weight (BW, g), body weight gain (ΔBWG), blood pressure measured by tail cuff pressure (Blood Pressure, mmHg), and 24 h urinary albumin excretion rate (24 h-UAE, mg/24 h). The number of animals studied per group was, respectively, Control ♂: *N* = 10; Control ♀: *N* = 10; NAME ♂: *N* = 7; NAME ♂: *N* = 9. One-way analysis of variance with pairwise comparisons according to the Newman-Keuls method was used to compare experimental groups: ^a^*p* < 0.05 versus Control ♂; ^b^*p* < 0.05 versus Control ♀; ^c^*p* < 0.05 versus NAME ♂.
